# Assessing chemotherapy-induced peripheral neuropathy with patient reported outcome measures: a systematic review of measurement properties and considerations for future use

**DOI:** 10.1007/s11136-022-03154-7

**Published:** 2022-05-21

**Authors:** Tiffany Li, Susanna B. Park, Eva Battaglini, Madeleine T. King, Matthew C. Kiernan, David Goldstein, Claudia Rutherford

**Affiliations:** 1 Faculty of Medicine and Health, School of Medical Sciences, Brain and Mind Centre, The University of Sydney, Camperdown, Sydney, NSW 2050 Australia; 2 Prince of Wales Clinical School, University of New South Wales, Kensington, Australia; 3 Faculty of Science, School of Psychology, Sydney Quality of Life Office, The University of Sydney, Sydney, Australia; 4 Prince of Wales Hospital, Randwick, Australia; 5 Faculty of Medicine and Health, Cancer Nursing Research Unit, The University of Sydney, Sydney, Australia

**Keywords:** Chemotherapy-induced peripheral neuropathy, Patient reported outcome measures, Cancer survivors, Measurement properties, Systematic review, Psychometric evaluation

## Abstract

**Purpose:**

Chemotherapy-induced peripheral neuropathy (CIPN) is a common toxicity of cancer treatment, with potential to significantly impact cancer survivors’ long-term quality of life. Patient reported outcome measures (PROMs) are increasingly utilised to evaluate CIPN. However, guidance remains lacking on how to identify fit for purpose PROMs with considerations necessarily differing when used in various research and in-clinic contexts. This study aimed to evaluate evidence about CIPN PROMs measurement properties and propose considerations to optimize CIPN PROM selection for each purpose.

**Methods:**

A systematic review was conducted to identify literature assessing measurement properties of CIPN PROMs. These were evaluated against Consensus-based Standards for the selection of health Measurement Instruments (COSMIN) criteria and International Society for Quality of Life minimum standards. Risk of Bias (RoB) was assessed using the COSMIN RoB checklist.

**Results:**

Thirty-nine papers evaluating measurement properties of 13 PROMs were included. The European Organization for Research and Treatment of Cancer Quality of Life Chemotherapy-Induced Peripheral Neuropathy Questionnaire (QLQ-CIPN20) and Functional Assessment of Cancer Therapy/Gynecologic Oncology Group-Neurotoxicity (FACT/GOG-Ntx) were the most commonly investigated PROMs and had the most measurement properties meeting established criteria.

**Conclusion:**

The use of the QLQ-CIPN20 and FACT/GOG-Ntx to assess CIPN in research settings has the most supporting evidence. However other considerations including study aims, endpoints and target population also factor into PROM selection and need to be considered more often when determining the most suitable outcome measure. Evidence of CIPN PROMs use in clinical practice is limited and their adoption to individual-patient level management requires more evaluation.

**Supplementary Information:**

The online version contains supplementary material available at 10.1007/s11136-022-03154-7.

Chemotherapy-induced peripheral neurotoxicity (CIPN) is a debilitating adverse side effect of chemotherapy treatment and is a common cause of treatment dose modification [1]. CIPN is characterised by peripheral nerve dysfunction leading to numbness, tingling, weakness and pain in the hands and feet that produces deficits in balance, gait and fine motor function [2]. Long-lasting CIPN has been demonstrated to continue affecting physical function post-treatment [3], negatively impacting on cancer survivors’ health-related quality of life (QOL) [4].

There remains a lack of consensus on the ideal standardised assessment tool for evaluating CIPN in research settings and clinical practice. Currently, the National Cancer Institute Common Terminology Criteria for Adverse Events (NCI-CTCAE) neuropathy subscale is the most ubiquitous measure of CIPN, despite low inter-rater reliability and low sensitivity to change [5, 6]. Due to these limitations, numerous measures have been proposed to assess CIPN, with approaches including assessing functional impairment, clinical and neurological examination and patient reported questionnaires [7].

Patient reported outcome measures (PROMs) are increasingly recognised as a valuable tool to collect CIPN symptom information [8]. CIPN PROMs are predominantly used in research settings to characterise the natural history of neuropathy development and recovery, and as endpoint measures in CIPN treatment and prevention studies. PROMs provide a meaningful measure of CIPN from the patient perspective, essential to capture symptom severity and impact on the patient’s activities of daily living. Such information is also valuable in clinical practice, where treatment modification is often guided by CIPN symptom expression and severity. However, little evidence exists on the use of CIPN PROMs in clinical practice.

While various PROMs have been developed to assess CIPN [8], it is important to investigate whether these measures satisfy established criteria for measurement properties including validity, reliability, responsiveness and interpretability [9]. Responsiveness is a particularly key measurement property in CIPN outcome assessment as symptoms develop progressively during neurotoxic chemotherapy administration and it is crucial to be able to detect early nerve dysfunction. This review focusses on PROM properties and selection for research settings, but also highlights considerations for future work needed to employ these tools in routine clinical practice.

Our aim was to systematically review the quality of data available on measurement properties of CIPN PROMs. Accordingly, we included studies addressing psychometric properties of CIPN PROMs and PROMs without any studies concerning validation or psychometric properties were outside the scope of this review. We summarised and highlighted gaps in the evidence, and recommended key considerations when selecting a PROM for use in future research studies and in clinical practice.

## Method

This systematic review was registered with the International Prospective Register for Systematic Reviews (PROSPERO) ID CRD42020210405 with no deviations from the original registered protocol and followed the Preferred Reporting Items for Systematic Reviews and Meta-analyses (PRISMA) guidelines [10].

### Literature search

A database search was undertaken on 29 September 2020 in CINAHL, Cochrane, Embase, PubMed, Scopus and PROQOLID for original research papers assessing at least one measurement property of a PROM used to assess CIPN published from database inception to 29 September 2020. The search included terms for “patient reported outcome measures”, “measurement properties” and “chemotherapy-induced peripheral neuropathy”. A detailed search strategy is available in the online supplement (S1). Searches were limited to the English language and full-text manuscripts. The construct of interest was to evaluate PROMs which assessed core CIPN symptom manifestations. Accordingly, PROMs which only evaluated neuropathic pain were excluded, as neuropathic pain occurs only in a proportion of patients with CIPN [11]. Outcome measures were also excluded if they were not purely questionnaire based (e.g., included neurological assessments), were designed for a paediatric cohort, or were reported by clinicians. Conference proceedings, editorials and reviews were also excluded. Additional searches of review articles and references of included manuscripts were undertaken to ensure comprehensive coverage of CIPN PROMs. An updated literature search was performed on 16 December 2021 on PubMed including the validated PubMed search filter developed by COSMIN researchers [12] to identify additional papers.

### Screening

Manuscript screening was completed using the online software Covidence. Retrieved studies were checked for duplicates and titles and abstracts were screened against the eligibility criteria. Full-text for potentially relevant studies, or where eligibility could not be confirmed from the abstract, were obtained and assessed for inclusion in this review. All screening was completed by two reviewers independently (TL and EB), with discrepancies resolved through discussion.

### Data extraction

Information from each publication was extracted including the study design, sample size, neurotoxic agents received, PROM investigated, measurement properties assessed and results of psychometric property evaluation.

### Appraisal of study quality and evaluation of measurement property results

The COnsensus based Standards for the selection of health Measurement INstruments (COSMIN) guidelines were utilised to assess the methodological quality of each included study and to apply criteria to grade measurement properties of included PROMs [9, 13, 14]. According to these guidelines, eight measurement properties were evaluated: (1) content validity, (2) structural validity, (3) internal consistency reliability (4) cross-cultural validity/measurement invariance, (5) test–retest reliability, (6) measurement error (7) construct validity and (8) responsiveness. COSMIN definitions of each measurement property and associated criteria are available in the online supplement (S2). Criterion validity was not assessed as there is currently no agreed gold-standard in CIPN testing [5].

The COSMIN Risk of Bias (ROB) checklist [9] was used to assess the methodological quality of studies included in the systematic review. The checklist consists of a four-point grading system to rate standards set for each measurement property per study as outlined in [9], using the ratings of ‘very good’, ‘adequate’, ‘doubtful’ or inadequate’.

The overall quality of evidence for each measurement property for each PROM was graded using the Grading of Recommendations Assessment, Development, and Evaluation (GRADE) approach [13]. Four factors are taken into consideration (ROB, inconsistency (consistency of results between studies), imprecision (total sample size of available studies) and directness (evidence from population of interest)), with the overall quality of evidence graded as ‘high’, ‘moderate’, ‘low’ or ‘very low’ [1] (available in Tables 2 and 3).

Results of each measurement property analysis from each study were evaluated against the criteria for good measurement properties [13] detailed in the online supplement S2, and given a positive (+), negative (−) or indeterminate rating (?) to determine if the property satisfied minimum requirements. The criteria included minimum requirements for structural validity (confirmatory factor analysis (CFA): comparative fit index (CFI) > 0.95 or root mean square error of approximation (RMSEA) < 0.06), internal consistency reliability (sufficient structural validity and Cronbach’s alpha ≥ 0.70 for each unidimensional scale or subscale), reliability (intraclass correlation coefficient (ICC) ≥ 0.70), measurement error (smallest detectable change (SDC) or limits of agreement (LoA) < minimal important change (MIC)), construct validity (the result is in accordance with the hypothesis), cross-cultural validity/measurement invariance (no important difference between group factors), and responsiveness (the result is in accordance with hypothesis). Measurement properties were evaluated for each PROM, and not for subscales within each PROM as these outcome measures are typically used in their entirety. The results of each individual measurement property analysis from each study were pooled and summarised to produce an overall rating for each measurement property per PROM and rated as ‘sufficient (+)’, ‘insufficient (−)’, ‘inconsistent (±)’ or ‘indeterminate (?)’ (available in Table 3).

Content validity was evaluated based on PROM development studies, content validity studies as well reviewer (TL) rating of PROM content, investigating the outcome measure’s relevance, comprehensiveness and comprehensibility, according to the COSMIN guidelines [14]. If there was high quality evidence for insufficient content validity, the PROM would not be further assessed and should not be recommended for use.

An additional measurement property, interpretability, was also evaluated in line with the International Society for Quality of Life (ISOQOL) recommendations for ensuring interpretability of PROMs [15]. This was assessed by investigating whether a guide to meaningful interpretation of scores (including what comprises a clinically relevant change in scores) was available and graded as ‘available’/‘not available’. Interpretability of a PROM is commonly addressed by estimating minimally important difference (MID), defined as the smallest difference in scores within-person that may impact the patient’s care [16]. Ideally, MIDs are estimated by ‘anchor-based’ methods that apply various relevant patient-rated, clinician-rated, or disease-specific variables to ‘anchor’ PROM data. ‘Distribution-based’ methods using only the PROM data, such as standard deviation and standard error of measurement, provide supportive estimates [17], but are considered inferior to anchor-based methods as these provide insights into the clinical relevance of differences [18].

## Results

**Fig. 1:**
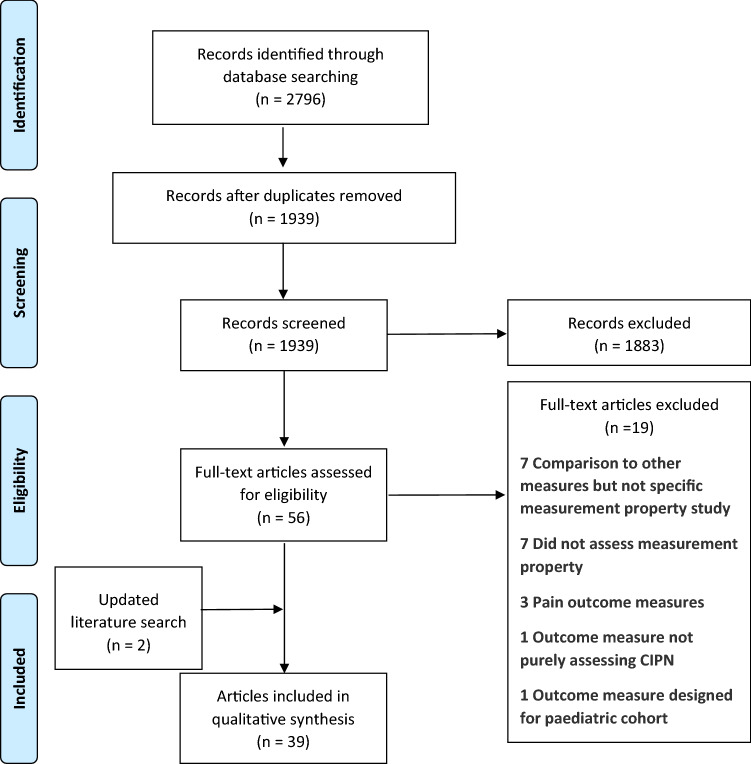
PRISMA flow diagram of record selection process

A total of 2797 papers were retrieved, 1939 after duplicates were removed. A further 1883 citations were removed following abstract screening, with 56 full-text papers obtained. Nineteen were excluded for not meeting inclusion criteria. Updated literature search identified two additional papers, for total of 39 included papers, evaluating 13 PROMs (Fig. 1); Table 1 summarises these PROMs and domains assessed. Twenty-five studies included a cross-sectional cohort, and 17 studies included a longitudinal cohort. Study sample sizes ranged from 24 to 1008, mean age ranged 45.2–64.0 years, and majority of participants were female (30/39 studies had > 50% female participants). Most studies recruited patients receiving multiple neurotoxic chemotherapies (31/39 studies). Commonly included neurotoxic agents were taxane-based (35 studies) or platinum-based (27 studies), bortezomib (15 studies), vinca-alkaloids (13 studies), and immunomodulatory drugs (eight studies).

**Table 1:** Summary of PROMs identified in systematic review

PROM name	Abbreviation	Number of items	Scales/domains assessed	Cumulative sample size	References
European Organization for Research and Treatment of Cancer Quality of Life Chemotherapy-Induced Peripheral Neuropathy Questionnaire	EORTC QLQ-CIPN20	20 (15- and 16-item versions also investigated)	Sensory, motor and autonomic neuropathy	5252 patients 595 controls	[19, 20, 25–35]
Functional Assessment of Cancer Therapy/Gynecologic Oncology Group—Neurotoxicity	FACT/GOG-Ntx	11 13 (Oxaliplatin-treatment specific version)	Sensory, motor and auditory neuropathy and dysfunction	1308 patients 206 controls	[32, 36–38, 40– 42, 66, 67]
Chemotherapy-Induced Peripheral Neuropathy Assessment Tool	CIPNAT	50	Symptom experience and interference	884 patients 40 controls	[44–46]
Treatment-induced Neuropathy Assessment Scale	TNAS	Version 1- 11-items Version 2- 13-tems Version 3- 9-items	Sensory symptoms and interference	663 patients	[47–49]
Comprehensive Assessment Scale for Chemotherapy-Induced Peripheral Neuropathy	CAS-CIPN	15	Threatened interference with daily life by negative feelings Impaired hand fine motor skills Confidence in choice of treatment/management Dysesthesia of palms and soles	327 patients	[68]
Indication for CTC Grading of Peripheral Neuropathy Questionnaire	ICPNQ	17	Sensory, motor and autonomic neuropathy	156 patients	[69]
4-Item Neurotoxicity Survey- Korean Version	K-NTX-4	4	Sensory neuropathy	237 patients	[43]
Chemotherapy-Induced Peripheral Neuropathy Rasch-Built Overall Disability Scale	CIPN R-ODS	28	Activity limitation and participation restriction due to CIPN	281 patients	[60]
Oxaliplatin-Associated Neurotoxicity Questionnaire/ Chemotherapy-Induced Neurotoxicity Questionnaire	OANQ/CINQ	29	Upper extremity, lower extremity and oral/facial symptoms	112 patients	[70, 71]
National Cancer Institute Patient-Reported Outcome Common Terminology Criteria for Adverse Events- Numbness & tingling	PRO-CTCAE	2	Severity and impact of CIPN	1584 patients	[30, 51–53]
Patient Neurotoxicity Questionnaire	PNQ	2	Sensory and motor disturbances	300 patients	[72]
10- Point Visual Analogue Scale	10-Point VAS	1	Pain/Numbness	93 patients	[73]
Chemotherapy-Induced Peripheral Neuropathy Self Check Sheet	CIPN Self check sheet	14	Upper limbs, lower limbs, pain, limitations in activities of daily living	77 patients	[74]

Of the 13 PROMs identified, the European Organization for Research and Treatment of Cancer Quality of Life Chemotherapy-Induced Peripheral Neuropathy Questionnaire (EORTC QLQ-CIPN20) and Functional Assessment of Cancer Therapy/Gynecologic Oncology Group Neurotoxicity subscale (FACT/GOG-Ntx) were the most investigated, with 13 and nine studies assessing their measurement properties respectively. Measurement properties of the Chemotherapy-Induced Peripheral Neuropathy Assessment Tool (CIPNAT), Treatment-induced Neuropathy Assessment Scale (TNAS) and National Cancer Institute Patient-Reported Outcome Common Terminology Criteria for Adverse Events- Numbness & tingling (PRO-CTCAE) were investigated in two to four studies while the remaining nine PROMs had measurement properties reported in only one study.

### COSMIN assessment of PROMs

Extracted measurement property results for each study are available in the online supplement (S3). Cross-cultural validity and measurement error were not addressed for any PROMs, and only two PROMs evaluated interpretability. Content validity evaluation for each PROM is detailed in Table 2. There was no high-quality study evidence that any included PROMs had insufficient content validity, so remaining properties of all PROMs were assessed. A summary of the overall rating and quality of evidence for other measurement properties per PROM is provided in Table 3. A number of PROMs (QLQ-CIPN20, FACT/GOG-Ntx and TNAS) have multiple versions, however studies investigating the measurement properties of the different versions of each PROM were combined in the results tables, but are explained independently in the text results. Structural validity was assessed in four PROMs and were all evaluated on the dimensionality of the entire PROM. Responsiveness of PROMs were evaluated in 14 studies by examining score changes over time. Notably, the QLQ-CIPN20 and FACT/GOG-Ntx met the highest number of established criteria compared to other PROMs, and are discussed in detail below.

**Table 2:** Content validity rating of the included PROMs

PROM		Relevance	Comprehensiveness	Comprehensibility	Overall content validity rating	Overall quality of evidence
EORTC-QLQ-CIPN20 (Including reduced versions)	Overall Rating	+	+	+	Sufficient (+)	Moderate
	Reviewer Rating	+	+	+		
FACT/GOG Ntx-13 (Including reduced versions)	Overall Rating	+	?	?	Sufficient (+)	Very good
	Reviewer Rating	+	+	+		
CIPNAT	Overall Rating	+	+	+	Sufficient (+)	Very low
	Quality of evidence	+	+	+		
TNAS	Overall Rating	+	?	+	Sufficient (+)	Moderate
	Reviewer Rating	+	+	+		
CAS-CIPN	Overall Rating	?	?	?	Insufficient (−)	Very low
	Reviewer Rating	−	−	−		
ICPNQ	Overall Rating	?	?	?	Sufficient (+)	Very low
	Reviewer Rating	+	−	+		
K-NTX-4	Overall Rating	?	?	?	Sufficient (+)	Very low
	Reviewer Rating	+	−	+		
R-ODS	Overall Rating	?	?	?	Insufficient (−)	Moderate
	Reviewer Rating	−	−	+		
OANQ/CINQ	Overall Rating	?	?	?	Sufficient (+)	Very low
	Reviewer Rating	+	−	+		
PRO-CTCAE	Overall Rating	?	?	?	Sufficient (+)	Very low
	Reviewer Rating	±	−	+		
PNQ	Overall Rating	?	?	?	Insufficient (−)	Very low
	Reviewer Rating	−	−	+		
10-Point VAS	Overall Rating	?	?	?	Insufficient (−)	Very low
	Reviewer Rating	−	−	−		
CIPN Self check sheet	Overall Rating	?	?	?	Insufficient (−)	Very low
	Reviewer Rating	+	−	−

**Table 3:** Overall summary of results and quality of evidence

Measurement property	Summary of pooled result	Overall rating	Overall quality of evidence
Structural validity			
EORTC-QLQ-CIPN20 (Including reduced versions)	Confirmatory factor analysis did not support 3-factor structure of PROM	Insufficient (−)	High
FACT/GOG Ntx-13 (Including reduced versions)	Confirmatory factor analysis did not support 4-factor structure of PROM	Insufficient (−)	High
CIPNAT	Exploratory and confirmatory factor analysis	Insufficient (−)	Very low; same sample used for EFA and CFA
CAS-CIPN	Exploratory and confirmatory factor analysis	Insufficient (−)	Very low; same sample used for EFA and CFA
Internal consistency reliability			
EORTC-QLQ-CIPN20	Summarised Cronbach alpha = 0.73–0.91; total sample size = 2208; structural validity overall rating insufficient	Insufficient (−)	High
FACT/GOG Ntx-13	Summarised Cronbach alpha = 0.82–0.91; total sample size > 994; structural validity overall rating insufficient	Insufficient (−)	High
CIPNAT	Summarised Cronbach alpha = 0.81–0.97; total sample size = 735; structural validity overall rating insufficient	Insufficient (−)	High
TNAS	Summarised Cronbach alpha = 0.80–0.90; total sample size = 469; structural validity/unidimensionality not investigated	Insufficient (−)	High
CAS-CIPN	Cronbach alpha = 0.83; sample size = 327; structural validity overall rating insufficient	Insufficient (−)	High
ICPNQ	Cronbach alpha = 0.61–0.84; sample size = 156; structural validity/unidimensionality not investigated	Insufficient (−)	High
K-NTX-4	Cronbach alpha = 0.89; sample size = 237; structural validity/unidimensionality not investigated	Insufficient (−)	High
R-ODS	Pearson separation index = 0.92; sample size = 281; structural validity/unidimensionality not investigated	Insufficient (−)	High
OANQ/CINQ	Cronbach alpha = 0.84–0.94; sample size = 23; structural validity/unidimensionality not investigated	Insufficient (−)	Low; low sample size
Test–retest reliability			
EORTC-QLQ-CIPN20 (Including reduced versions)	Correlation between tests = 0.73–0.86	Sufficient (+)	Moderate; unsure if patients changed neuropathy status between tests
CIPNAT	Correlation between tests = 0.89–0.93	Sufficient (+)	High
TNAS	ICC = 0.97	Sufficient (+)	Very low; test conditions were not similar between tests
ICPNQ	ICC = 0.83	Sufficient (+)	Moderate; unsure if patients changed neuropathy status between tests
K-NTX-4	ICC = 0.84	Sufficient (+)	Moderate; unsure if patients changed neuropathy status between tests
R-ODS	ICC or weighted Kappa not reported	Insufficient (−)	Low
OANQ/CINQ	ICC for each item ranged 0.1–1.0	Sufficient (+)	Very low; short re-test interval (1 h) and low sample size (*n* = 24)
PRO-CTCAE	ICC for severity is 0.8, ICC for interference is 0.55	Sufficient (+) for severity Insufficient (−) for interference	Low; only assumable that test conditions similar, low sample size (*n* = 80)
Construct validity			
EORTC-QLQ-CIPN20 (Including reduced versions)	14/15 hypotheses supported	Sufficient (+)	High
FACT/GOG Ntx-13 (Including reduced versions)	7/8 hypotheses supported	Sufficient (+)	High
CIPNAT	3/3 hypotheses supported	Sufficient (+)	High
CAS-CIPN	1/1 hypotheses supported	Sufficient (+)	Very low; due to risk of bias and low sample size
ICPNQ	2/2 hypotheses supported	Sufficient (+)	High
K-NTX-4	0/1 hypotheses supported	Insufficient (−)	Low; insufficient information on comparator instrument
PRO-CTCAE	4/4 hypotheses supported	Sufficient (+)	High
PNQ	1/1 hypotheses supported	Sufficient (+)	High
CIPN self check sheet	1/1 hypotheses supported	Sufficient (+)	Moderate; low sample size
Responsiveness			
EORTC-QLQ-CIPN20 (Including reduced versions)	3/3 hypotheses supported	Sufficient (+)	High
FACT/GOG Ntx-13 (Including reduced versions)	6/7 hypotheses supported	Sufficient (+)	High
TNAS	1/2 hypotheses supported	Insufficient (−)	High
PNQ	1/1 hypotheses supported	Sufficient (+)	High
10-Point VAS	1/1 hypotheses supported	Sufficient (+)	Very low; due to risk of bias and low sample size

### EORTC QLQ-CIPN20

The QLQ-CIPN20 is a 20-item questionnaire designed to supplement EORTC core Quality of Life questionnaire, EORTC QLQ-C30 (https://qol.eortc.org/) [19]. It is one of the most widely used PROMs for assessing CIPN in clinical research (including observational studies and CIPN treatment and prevention trials), with evidence of feasibility for multi-cultural use in large multi-site studies [20–24]. It was designed to assess three aspects of neuropathy in corresponding subscales: sensory, motor, and autonomic [19]. Patients indicate the degree to which each symptom (“item”) is experienced in the last 7 days using a four-point scale (1 = Not at all, 2 = A little bit, 3 = Quite a bit, 4 = Very much). The three subscales are each calculated as the sum of component items, linearly converted to a 0–100 scale, with higher scores indicating greater symptom burden [19].

#### PROM development and content validity

The QLQ-CIPN20 was developed in four phases [19]: literature search and selection of key issues by 15 health care professionals and 68 patients, operationalisation and editing based on feedback, pre-testing the provisional measure on 44 patients, and international field-testing. Content validity was assessed by CIPN experts and patients [25, 26] with authors suggesting acceptable content validity using content validity indices (CVI, > 0.8). Although CVI is not a COSMIN criteria for content validity, it has been used in a number of content validity studies to investigate this measurement property.

#### Evaluation of internal structure

Structural validity and consequently, internal consistency reliability, were not demonstrated for hypothesised subscales, with confirmatory factor analyses (CFA) consistently demonstrating poor model fit for the 3-factor structure [19, 26–34]. Exploratory factor analysis (EFA) suggested a 2-factor structure (upper vs lower limb symptoms) [26], but this was subsequently demonstrated to have poor model fit [29, 33].

#### Other measurement properties

Test–retest reliability, responsiveness and construct validity for the QLQ-CIPN20 have met acceptable criteria in multiple studies [20, 26–30, 32, 33] (Table 3). Only one study estimated MIDs for the QLQ-CIPN20, using distribution-based methods [35].

The QLQ-CIPN20 has been translated into numerous languages, with psychometric properties assessed for Arabic [26], Korean [28], and Chinese versions [32]. However, cross-cultural and linguistic validity has not been assessed in any translated versions, so equivalence across versions has not been determined.

#### Reduced versions of QLQ-CIPN20

Reduced versions of the QLQ-CIPN20 including the 15- and 16-item variant have been proposed after removing questions with low item-total correlation (items addressing hearing loss, orthostatic hypotension, blurred vision, erectile dysfunction and difficulty driving) [25, 27]. Cognitive interviews evaluating content validity on the 16-item version found that altered wording could improve patient comprehension of certain items (including “loss of feeling” added to “numbness”, rewording “shooting or burning pain in hands/feet” to “is the numbness/tingling in your (fingers or hands)/(toes or feet) painful”) [25]. Evidence supported test–retest reliability, construct validity and responsiveness for the reduced versions, with acceptable Cronbach’s alpha (*α* ≥ 0.7) for the overall PROM [25, 29]. However, CFA did not demonstrate structural validity and consequently internal consistency, and further work will be required to ascertain the unidimensionality of the PROM and its internal structure.

### FACT/GOG-Ntx

#### PROM development and content validity

The Functional Assessment of Chronic Illness Therapy (https://www.facit.org/) group collaborated with the Gynecologic Oncology Group (GOG) to develop the FACT/GOG-Ntx. The original 11-item measure (FACT/GOG-Ntx11) was designed to assess the severity and impact of CIPN on patients’ lives [36] related to sensory, motor and auditory neuropathy and dysfunction [37]. Two items were subsequently added to assess cold-induced neuropathy specific to oxaliplatin treatment [38], forming the FACT/GOG-Ntx13. Each item is scored on a five-point scale (0 = Not at all, 1 = A little bit, 2 = Somewhat, 3 = Quite a bit, 4 = Very much). Following the FACIT group’s scoring convention, items scores are reversed with higher total scores indicating better outcomes [39]. Content validity has been investigated [40] using concept elicitation and cognitive debriefing methods; content validity was found to be strong for eight items, moderate for three and weak for two.

#### Evaluation of internal structure

EFA and CFA have been undertaken to investigate the structural validity of the FACT/GOG-Ntx11. CFA did not support a four-factor structure [40, 41]. EFA identified a two-factor structure (hands and feet) [41], but this was not subsequently supported [42], with a further EFA suggesting that the FACT/GOG-Ntx13 was unidimensional [38] although this would need to be further confirmed with a CFA. According to COSMIN guidelines [13], despite the PROM showing acceptable Cronbach’s alpha, internal consistency reliability could not be confirmed due to lack of evidence for structural validity.

#### Other measurement properties

Construct validity and responsiveness have also been supported in high quality studies (Table 3). Test–retest reliability has not been assessed for the FACT/GOG-Ntx. The FACT/GOG-Ntx has been translated by FACIT into multiple languages. However, our search found no cross-cultural validation studies.

There has been one FACT/GOG-Ntx MID estimation study to date providing guidance for interpretability [41]. However, these were determined with distribution-based methods, and have similar limitations to MID estimates for the QLQ-CIPN20 noted above.

#### Reduced versions of FACT/GOG-Ntx

A short four-item version of the FACT/GOG-Ntx, the NTX-4, assessing numbness, tingling and discomfort in the hands and feet has met minimum criteria for test–retest reliability [43]. Construct validity and structural validity were assessed but did not meet minimum criteria [43]. No other measurement properties have been assessed for the NTX-4.

### Other CIPN PROMs

Several other PROMs have been used CIPN research, although their measurement properties have not been as comprehensively investigated.

The CIPNAT is a 50-item PROM, designed to assess neuropathic symptoms and their interference with daily activities [44]. Measurement properties were assessed in three studies [44–46]. Content validity was investigated by health professionals and CIPN patients; CVI met acceptable index according to authors (> 0.8), but methods for assessing relevance and comprehensiveness of the PROM’s content were inadequately described. Test–retest reliability and construct validity met established criteria (Table 3).

The TNAS was developed to assess CIPN in neurotoxic chemotherapy-treated cohorts [47]. This PROM has several versions (11-item, 13-item and 9-item versions) [47–49]. The measurement properties of the most recent TNAS version (v3, 9-item version) were investigated in one study [48], finding that test–retest reliability met established criteria.

The PRO-CTCAE was developed to evaluate a broad range of toxicities in cancer patients, complementing the original clinician-reported NCI-CTCAE measures of adverse events [50]. The PRO-CTCAE sensory neuropathy subscale is a 2-item measure: the first assesses the severity of numbness/tingling in the hands and feet (0 = none to 4 = very severe) and the second assesses the degree which numbness/tingling interferes with usual daily activities (0 = not at all to 4 = very much). Although this PROM was developed and endorsed by the NCI, only test–retest reliability and construct validity have been investigated and determined to meet minimum criteria [30, 51–53].

Other PROMs including the CAS-CIPN, ICPNQ, R-ODS, OANQ, PNQ, 10-Point VAS, CIPN Self check sheet and CINQ (see Table 1 for abbreviations) have also been designed to assess CIPN, but their measurement properties have not been comprehensively evaluated.

## Discussion

This systematic review identified available evidence about the measurement properties of 13 CIPN PROMs from 39 studies. We critically evaluated these measurement properties against COSMIN criteria and identified gaps in evidence. The QLQ-CIPN20 and FACT/GOG-Ntx were the most commonly investigated PROMs and met the highest number of measurement property criteria. We discuss these key PROMs and provide considerations for CIPN PROM selection for future use.

### QLQ-CIPN20 and FACT/GOG-Ntx: considerations for use

The QLQ-CIPN20 and FACT/GOG-Ntx have the most extensive evidence supporting their content coverage and measurement properties. Important measurement properties including responsiveness and construct validity have been confirmed in high quality studies for both PROMs, demonstrating that these measures are able to detect change over time and are assessing the appropriate construct of interest (i.e., correlation with other CIPN outcome measures including the Total Neuropathy Score, NCI-CTCAE, WHO-CIPN). However, MID estimation studies used solely distribution-based methods [35, 41]. Future MID estimation studies should use anchor-based methods to facilitate interpretation and optimise the utility of these PROMs.

PROM content is an important consideration when selecting a CIPN PROM. There are differences between the QLQ-CIPN20 and FACT/GOG-Ntx in the concepts assessed by each PROM. For example, the QLQ-CIPN20 more thoroughly explores functional impacts of CIPN, with questions including “problems standing or walking due to difficulty feeling the ground under your feet” and “difficulty holding a pen which has made writing difficult”. There are also more subtle differences, e.g., the QLQ-CIPN20 asks specifically about “burning or shooting pain in the hands/feet”, while the FACT/GOG-Ntx is less specific: “discomfort in the hands/feet”. Further, both instruments fail to capture some impacts of CIPN. For example, sleep disturbance, balance impairment and reduced physical activity have been identified as significant distal outcomes resulting from CIPN [54] but are not captured by either PROM. Accordingly, while the QLQ-CIPN20 and FACT/GOG-Ntx are psychometrically sound and adequately assess proximal outcomes of CIPN (e.g., symptoms), they may not capture all downstream impacts of neurotoxicity that are relevant to patients’ daily function and QOL. If assessment of these impacts is a goal of a research study, supplementing these symptom-focussed measures with another PROM which more comprehensively assesses the impact of neuropathy on functioning should be considered.

A further consideration is the internal structure of the PROM. Although both the QLQ-CIPN20 and FACT/GOG-Ntx were initially developed to evaluate neuropathy via distinct subscales (QLQ-CIPN20: sensory, motor, autonomic; FACT/GOG-Ntx: sensory, motor, auditory, dysfunction), assessment of structural validity has not supported these subscales. This suggests that capturing the range of symptoms and impacts of CIPN in one total score is more sound psychometrically rather than separating symptoms into subscales [35]. In part, this may be due to miscategorised symptoms. For example, “difficulty manipulating small objects with your hands (e.g., fastening buttons)” is considered a symptom of motor neuropathy in the QLQ-CIPN20 subscale, although patients with numbness in the fingertips (indicating sensory neuropathy) also report difficulty with these tasks. The lack of subscales does not necessarily undermine the utility of these instruments—however it may be a consideration if the aim of PROM use is to examine or compare specific neuropathy symptom categories. However confirmatory analyses need to be undertaken to verify unidimensionality and confirm internal consistency reliability for CIPN PROMs.

A common and important purpose for these PROMs is as clinical trial outcome measures in CIPN prevention and treatment trials. Responsiveness is a key consideration for this purpose. While studies have demonstrated responsiveness for the QLQ-CIPN20 and FACT/GOG-Ntx in detecting CIPN symptom development, a clinical trial outcome measure also needs to be able to detect improvement in patients with established CIPN, although this has not yet been formally established for these two PROMs. However, these PROMs have been utilised in a number of clinical trials, some of which have demonstrated some CIPN symptom improvement, which may suggest responsiveness [22]. Accordingly, shorter versions of the QLQ-CIPN20 and FACT/GOG-Ntx (CIPN8 and NTX-4) have also been adapted for use as endpoint measures in clinical trials [55, 56]. However, their measurement properties and suitability for this use have not been comprehensively assessed.

### Other CIPN PROMs

This review also identified a diverse range of other CIPN PROMs, which may be suited to particular applications but require further evidence to support their measurement properties. Of note, guidance for interpreting PROM scores has not been described for any CIPN PROMs other than QLQ-CIPN20 and FACT/GOG-Ntx. Further, responsiveness has only been investigated for limited CIPN PROMs.

Assessment of CIPN in specific settings may require quantification of symptom severity and functional impact. Accordingly, PROM content is an important feature which must be considered. Distal outcomes (e.g., sleep) that are not captured in the QLQ-CIPN20 and FACT/GOG-Ntx are generally not assessed by other PROMs. Both the CIPNAT [44–46] and the TNAS [47–49] incorporate impact of CIPN on patient function, including sleep and walking performance. However, responsiveness of the CIPNAT has not yet been evaluated and the 50-items may be overly burdensome, whereas properties of the TNAS have yet to be investigated by researchers external to the development group. The single-item 10-point VAS has the most limited content coverage, assessing only numbness and pain. Further, there has been minimal investigation of its measurement properties in CIPN research. Despite these major limitations, it has been used as an endpoint in numerous CIPN intervention trials [57, 58], perhaps because VAS scales are recommended for assessing chronic pain conditions and have been used extensively as clinical trial outcome measures [59]. We note that this is not a sound basis for judging its fitness for purpose in any particular CIPN setting.

The CIPN Rasch-built Overall Disability Scale (R-ODS) assesses the extent of disability resulting from CIPN [60] but not CIPN specific symptoms or their severity, so may have limited utility in research studies whose endpoint is CIPN. Furthermore, the R-ODS does not discriminate whether disability is due to CIPN or other treatment-related side-effects. These issues require consideration prior to PROM selection, depending on the purpose of use.

Another important consideration when deciding the most appropriate PROM for each application is the cohort in which the measurement property for the PROMs have been assessed, as different neurotoxic agents may result in varying CIPN presentations. Although most PROMs (including the QLQ-CIPN20 and FACT/GOG-Ntx) have been evaluated in multiple neurotoxic chemotherapy settings, some PROMs (such as the PNQ) have been evaluated in limited neurotoxic chemotherapy cohorts and their suitability in assessing neuropathy caused by other treatments needs to be considered.

### Fit for purpose PROMs in clinical practice and research settings

The selection of the most appropriate CIPN PROM will depend on purpose and setting (Table 4). It is therefore unlikely that a single ‘gold standard’ CIPN PROM will be found with utility across all settings and purposes. Investigators and clinicians first need to be clear on the purpose of PROM assessment and then identify the best available PROM for that purpose [61]. The majority of CIPN PROM development and evaluation has been conducted with a view to their use as outcomes measures in clinical studies with limited guidance available for PROM selection for clinical practice.

**Table 4:** Considerations for CIPN PROM selection in specific clinical practice and research settings

Setting	Considerations for PROM selection	Gaps in current research
Clinical practice		
Routine CIPN screening	Able to differentiate between patients with/without CIPN (footnote 1) Able to differentiate degrees of severity, with cut-points for severity levels: mild, moderate, severe Short form—minimal patient and clinician burden	Limited evidence for selection of CIPN PROMs as screening tool Suitable gold-standard measure of CIPN against which to assess the sensitivity and specificity of PROM thresholds
CIPN symptom monitoring	Responsive to symptom development and improvement Capture quality of life and functional impacts of CIPN Sufficiently reliable for individual-level use (footnote 2) Threshold for meaningful must be above bounds of measurement error (also footnote 2) Need to consider patient preferences and situation (during or post treatment, adjuvant or metastatic etc.) when making treatment decisions (e.g. to reduce dose due to increase in CIPN)	Limited evidence for CIPN PROMs as symptom monitoring tool Limited MIDs available and solely estimated in group-based settings
Research applications		
Observational studies	Responsive to symptom development and improvement Comprehensive content coverage tailored to study focus: symptom severity (proximal) and/or functional impacts (distal)	Lacking studies looking at PROM responsiveness for symptom improvement No PROMs with comprehensive distal functional impact coverage
Clinical trials—CIPN prevention/treatment trials	Responsive to symptom development and improvement Extent of content coverage may be limited by other trial factors, depending on study focus and other PROM measures required PROM must have interpretation guidelines, including anchor-based MID estimate	Robust MID guidelines for CIPN PROMs lacking Limited evidence examining PROM responsiveness for CIPN improvement
Clinical trial—adverse event outcome measure in cancer treatment trial	Responsive to symptom development and improvement Short, symptom-focused, quick to administer	PROM suitability for this setting not assessed

*PROM* patient reported outcome measure, *CIPN* chemotherapy-induced peripheral neuropathy, *MID* minimally important difference

1. Screening tools are typically not 100% accurate: a threshold must be set to identify probable cases of CIPN for further investigation. Inevitably there will be false positives and false negatives. The acceptable balance between false positives (‘false alarms’) and false negatives (missed cases of CIPN) must be found as this will impact on health service workload

2. Greater PROM reliability is needed at the individual-level (i.e. in clinical practice) than at the group-level (i.e. for research). Thresholds must be determined for the degree of observed change on a PROM scale that reliably reflects true change, and these must be above the bounds of measurement error to avoid ‘false-positive changes’ triggering unwarranted alerts and action in clinic

Even in research settings, PROM selection may differ depending on whether CIPN is the focus of the study, or one of many adverse events being monitored (Table 4). For example, in CIPN observational research studies, a comprehensive investigation may be appropriate, prioritizing responsiveness and detailed symptom coverage. Conversely, in clinical trials of cancer treatments where CIPN is one of many adverse events monitored, a brief, yet responsive PROM may be suitable. However, in CIPN intervention clinical trials, a PROM with detailed symptom coverage, used alongside other neurological or neurophysiological assessment tools may be more appropriate to assist understanding the physiology as well as patient perspective of neuropathy.

In routine clinical practice, other considerations may apply. For screening of CIPN symptom progression during treatment, shorter PROMs may be preferred; a Delphi survey found that instruments with more than three items had poor feasibility rating by clinicians for routine use [7]. The two-item PRO-CTCAE may be a good option for screening purposes [52]. However, screening tools are never 100% accurate; inevitably there will be false positives and false negatives [62]. It is important to consider this as screening of false positives will impact on health service workload. For monitoring patients with established CIPN, a more comprehensive PROM evaluating functional impacts of CIPN as well as symptom severity may be appropriate. When PROMs are used in clinic to monitor changes in CIPN, there may be additional considerations when translating individual-level data versus group-level data [62]. Appropriate MID cut points are needed to support the use of PROMs in clinical practice. Caution may be required to ensure that the threshold for meaningful change is relevant to individual patients to avoid ‘false-positive changes’ activating unnecessary action in clinic [62].

Another critical feature for use in clinic is acceptability from the patient perspective. A study of patient preferences for CIPN assessment found that patients prefer a comprehensive CIPN assessment including a physical test [63]. There are a range of different approaches to CIPN assessment beyond PROMs, including clinician-graded assessment (NCI-CTCAE), functional assessment (e.g., balance and fine motor function) and clinical neurological examination. Discordance between patient-reported symptoms and neurological signs of CIPN [64] suggest these approaches target different manifestations of CIPN, and should be used together to capture a comprehensive picture of CIPN. However, there needs to be balance between comprehensive assessment and appropriate resource use, depending on the setting and clinical population.

Clinician graded scales are currently the most commonly used CIPN assessment measures in both clinical practice and trials. There is substantial evidence of their significant limitations, including low inter-rater reliability and low sensitivity to change [5, 6], particularly when used without standardised training [20]. In contrast, PROMs are essential for patient-centred care, providing critical assessment of treatment toxicities such as CIPN, necessary to understand the impact of symptoms to the patient. However, it should be acknowledged that grading of CIPN via PROMs involves personalized assessments of symptom burden and tolerability, which are likely to differ among individuals [65]. It is important to consider this variability in clinical practice where treatment modification is often guided by toxicity grading. Further, existing CIPN PROMs have been developed and validated in group-level research settings, and care must be taken to not overinterpret results when adopting group-derived thresholds to individual-patient management [62]. This further underscores the need to define cut points to clarify MIDs for CIPN PROMs and develop guides for the use of CIPN PROMs in clinical practice. While clinicians typically report lower severity CIPN than severity reported directly by patients [5], unlike patients, clinicians have prior experience with the range of CIPN severity which can assist in benchmarking severity across individuals [65]. The development of guidance on how to combine patient and clinician perspectives in clinical practice is important and currently represents a gap in the field, limiting shared decision-making.

In summary, PROMs provide a valuable perspective of CIPN that assesses symptom burden and impact. The QLQ-CIPN20 and FACT/GOG-Ntx are the most psychometrically sound PROMs to date and are therefore recommended for use in research studies. Further work is required to evaluate measurement properties of other CIPN PROMs and investigate their fitness for purpose. Evidence for PROM use in clinical settings remains limited, and careful consideration is necessary when adopting and translating CIPN PROMs to an individual-based patient level. It is essential that characteristics of these PROMs including measurement properties, content coverage and comprehensiveness be carefully considered to ensure appropriateness for each specific research or clinical purpose. Ultimately, improving CIPN assessment is an important step towards enhanced recognition and management of toxicity, which is necessary to facilitate QOL in cancer survivors.

## Supplementary Information

Below is the link to the electronic supplementary material.

Electronic supplementary material 1 (DOCX 40 kb)

## Data Availability

Not applicable.
