# Supplementary material for: Assessing chemotherapy-induced peripheral neuropathy with patient reported outcome measures: a systematic review of measurement properties and considerations for future use
Source: Qual Life Res. 2022 May 21;31(11):3091–107. doi: 10.1007/s11136-022-03154-7 (PMC9546984; doi:10.1007/s11136-022-03154-7)
Supplement: Supplementary file 1 — Electronic supplementary material 1 (DOCX 40 kb) [file 11136_2022_3154_MOESM1_ESM.docx]

| **Database** | **Search terms** |
| --- | --- |
| CINAHL | Psychometric OR Valid* OR Reliab* OR Consist* OR Sensitiv* OR Responsiv* OR Development* OR Evaluation* OR Conceptual*  AND  Patient reported outcome* OR Patient report* OR Questionnaire* OR Patient reported outcome measure [MeSH term] OR Rating scale* OR Instrument* OR Survey* OR Measure*  AND  Chemotherap* OR Taxane OR Platinum OR Paclitaxel OR Docetaxel OR Abraxane OR Oxaliplatin OR Cisplatin OR Bortezomib OR Ixazomib OR Carfilzomib OR Thalidomide OR Lenalidomide OR Pomalidomide  AND  Chemotherapy induced peripheral neuropathy OR Neuropath* OR Neurotox* OR CIPN  Filters- Full text availability, English language, human studies |
| Cochrane Library | (Psychometric OR Valid* OR Reliab* OR Consist* OR Sensitiv* OR Responsiv* OR Development* OR Evaluation* OR Conceptual*)  AND  (Patient reported outcome* OR Patient report* OR Questionnaire* OR Patient reported outcome measure [exp] OR Rating scale* OR Instrument* OR Survey* OR Measure*)  AND  (Chemotherap* OR Antineoplastic Agents [exp] OR OR Taxane OR Platinum OR Paclitaxel OR Docetaxel OR Abraxane OR Oxaliplatin OR Cisplatin OR Bortezomib OR Ixazomib OR Carfilzomib OR Thalidomide OR Lenalidomide OR Pomalidomide)  AND  (Chemotherapy induced peripheral neuropathy OR Neuropath* OR Neurotox* OR CIPN OR Neurotoxicity Syndromes [MeSH term])  Filters: Full text availability, English language, human studies |
| Embase | Psychometric OR Valid* OR Reliab* OR Consist* OR Sensitiv* OR Responsiv* OR Development* OR Evaluation* OR Conceptual*  AND  Patient reported outcome* OR Patient report* OR Questionnaire* OR Patient reported outcome measure [MeSH term] OR Rating scale* OR Instrument* OR Survey* OR Measure*  AND  Chemotherap* OR Taxane OR Platinum OR Paclitaxel OR Docetaxel OR Abraxane OR Oxaliplatin OR Cisplatin OR Bortezomib OR Ixazomib OR Carfilzomib OR Thalidomide OR Lenalidomide OR Pomalidomide  AND  Chemotherapy induced peripheral neuropathy OR Neuropath* OR Neurotox* OR CIPN  Filters- Full text availability, English language, human studies |
| PubMed | "Psychometric"[Title/Abstract] OR "valid*"[Title/Abstract] OR "reliab*"[Title/Abstract] OR "consist*"[Title/Abstract] OR "sensitiv*"[Title/Abstract] OR "responsiv*"[Title/Abstract] OR "development*"[Title/Abstract] OR "evaluation*"[Title/Abstract] OR "conceptual*"[Title/Abstract]  AND  patient reported outcome*"[Title/Abstract] OR "patient report*"[Title/Abstract] OR "questionnaire*"[Title/Abstract] OR "patient reported outcome measures"[MeSH Terms] OR "patient reported outcome*"[Title/Abstract] OR "rating scale*"[Title/Abstract] OR "instrument*"[Title/Abstract] OR "survey*"[Title/Abstract] OR "measure*"[Title/Abstract]  AND  "chemotherap*"[Title/Abstract] OR "antineoplastic agents"[MeSH Terms] OR "antineoplastic agents"[MeSH Terms] OR "Taxane"[Title/Abstract] OR "Platinum"[Title/Abstract] OR "Paclitaxel"[Title/Abstract] OR "Docetaxel"[Title/Abstract] OR "Abraxane"[Title/Abstract] OR "Oxaliplatin"[Title/Abstract] OR "Cisplatin"[Title/Abstract] OR "Bortezomib"[Title/Abstract] OR "Ixazomib"[Title/Abstract] OR "Carfilzomib"[Title/Abstract] OR "Thalidomide"[Title/Abstract] OR "Lenalidomide"[Title/Abstract] OR "Pomalidomide"[Title/Abstract] OR "vinca alkaloid*"[Title/Abstract] OR "Vincristine"[Title/Abstract] OR "brentuximab vedotin"[Title/Abstract]  AND  "chemotherapy induced peripheral neuropathy"[Title/Abstract] OR "neuropath*"[Title/Abstract] OR "neurotox*"[Title/Abstract] OR "CIPN"[Title/Abstract] OR "neurotoxicity syndromes"[MeSH Terms]  Limits: Full text, human studies, English language |
| Scopus | TITLE-ABS (psychometric) OR TITLE-ABS (valid*) OR TITLE-ABS (reliab*) OR TITLE-ABS (consist*) OR TITLE-ABS (sensitiv*) OR TITLE-ABS (responsiv*) OR TITLE-ABS (development*) OR TITLE-ABS (evaluation*) OR TITLE-ABS (conceptual*)  AND  TITLE-ABS (patient AND reported AND outcome*) OR TITLE-ABS (patient AND report*) OR TITLE-ABS (questionnaire*) OR TITLE-ABS (rating AND scale*) OR TITLE-ABS (instrument*) OR TITLE-ABS (survey*) OR TITLE-ABS (measure*)  AND  TITLE-ABS (chemotherap*) OR TITLE-ABS (taxane) OR TITLE-ABS (platinum) OR TITLE-ABS (paclitaxel) OR TITLE-ABS (docetaxe) OR TITLE-ABS (abraxane) OR TITLE-ABS (oxaliplatin) OR TITLE-ABS (cisplatin) OR TITLE-ABS (bortezomib) OR TITLE-ABS (ixazomi ) OR TITLE-ABS (carfilzomib) OR TITLE-ABS (thalidomide) OR TITLE-ABS (lenalidomide) OR TITLE-ABS (pomalidomide) OR TITLE-ABS (vinca AND alkaloid*) OR TITLE-ABS (vincristine) OR TITLE-ABS (brentuximab AND vedotin)  AND  TITLE-ABS (chemotherapy AND induced AND peripheral AND neuropathy) OR TITLE-ABS (neuropath*) OR TITLE-ABS (neurotox*) OR TITLE-ABS (cipn)  Limits: Research article, English language |
| PROQOLID | Chemotherapy neuropathy |
| PubMed using validated filter developed by COSMIN researchers [12]  To ensure a comprehensive review an updated search was completed on 16 December 2021 using the COSMIN validated search filter | "chemotherapy induced peripheral neuropathy"[Title/Abstract] OR "neuropath*"[Title/Abstract] OR "neurotox*"[Title/Abstract] OR "CIPN"[Title/Abstract] OR "neurotoxicity syndromes"[MeSH Terms]  AND  "chemotherap*"[Title/Abstract] OR "antineoplastic agents"[MeSH Terms] OR "antineoplastic agents"[MeSH Terms] OR "Taxane"[Title/Abstract] OR "Platinum"[Title/Abstract] OR "Paclitaxel"[Title/Abstract] OR "Docetaxel"[Title/Abstract] OR "Abraxane"[Title/Abstract] OR "Oxaliplatin"[Title/Abstract] OR "Cisplatin"[Title/Abstract] OR "Bortezomib"[Title/Abstract] OR "Ixazomib"[Title/Abstract] OR "Carfilzomib"[Title/Abstract] OR "Thalidomide"[Title/Abstract] OR "Lenalidomide"[Title/Abstract] OR "Pomalidomide"[Title/Abstract] OR "vinca alkaloid*"[Title/Abstract] OR "Vincristine"[Title/Abstract] OR "brentuximab vedotin"[Title/Abstract]  AND  patient reported outcome*"[Title/Abstract] OR "patient report*"[Title/Abstract] OR "questionnaire*"[Title/Abstract] OR "patient reported outcome measures"[MeSH Terms] OR "patient reported outcome*"[Title/Abstract] OR "rating scale*"[Title/Abstract] OR "instrument*"[Title/Abstract] OR "survey*"[Title/Abstract] OR "measure*"[Title/Abstract]  AND  (instrumentation[sh] OR methods[sh] OR “Validation Studies”[pt] OR “Comparative Study”[pt] OR “psychometrics”[MeSH] OR psychometr*[tiab] OR clinimetr*[tw] OR clinometr*[tw] OR “outcome assessment (health care)”[MeSH] OR “outcome assessment”[tiab] OR “outcome measure*”[tw] OR “observer variation”[MeSH] OR “observer variation”[tiab] OR “Health Status Indicators”[Mesh] OR “reproducibility of results”[MeSH] OR reproducib*[tiab] OR “discriminant analysis”[MeSH] OR reliab*[tiab] OR unreliab*[tiab] OR valid*[tiab] OR “coefficient of variation”[tiab] OR coefficient[tiab] OR homogeneity[tiab] OR homogeneous[tiab] OR “internal consistency”[tiab] OR (cronbach*[tiab] AND (alpha[tiab] OR alphas[tiab])) OR (item[tiab] AND (correlation*[tiab] OR selection*[tiab] OR reduction*[tiab])) OR agreement[tw] OR precision[tw] OR imprecision[tw] OR “precise values”[tw] OR test-retest[tiab] OR (test[tiab] AND retest[tiab]) OR (reliab*[tiab] AND (test[tiab] OR retest[tiab])) OR stability[tiab] OR interrater[tiab] OR inter-rater[tiab] OR intrarater[tiab] OR intra-rater[tiab] OR intertester[tiab] OR inter-tester[tiab] OR intratester[tiab] OR intra-tester[tiab] OR interobserver[tiab] OR inter-observer[tiab] OR intraobserver[tiab] OR intra-observer[tiab] OR intertechnician[tiab] OR inter-technician[tiab] OR intratechnician[tiab] OR intra-technician[tiab] OR interexaminer[tiab] OR inter-examiner[tiab] OR intraexaminer[tiab] OR intra-examiner[tiab] OR interassay[tiab] OR inter-assay[tiab] OR intraassay[tiab] OR intra-assay[tiab] OR interindividual[tiab] OR inter-individual[tiab] OR intraindividual[tiab] OR intra-individual[tiab] OR interparticipant[tiab] OR inter-participant[tiab] OR intraparticipant[tiab] OR intra-participant[tiab] OR kappa[tiab] OR kappa’s[tiab] OR kappas[tiab] OR repeatab*[tw] OR ((replicab*[tw] OR repeated[tw]) AND (measure[tw] OR measures[tw] OR findings[tw] OR result[tw] OR results[tw] OR test[tw] OR tests[tw])) OR generaliza*[tiab] OR generalisa*[tiab] OR concordance[tiab] OR (intraclass[tiab] AND correlation*[tiab]) OR discriminative[tiab] OR “known group”[tiab] OR “factor analysis”[tiab] OR “factor analyses”[tiab] OR “factor structure”[tiab] OR “factor structures”[tiab] OR dimension*[tiab] OR subscale*[tiab] OR (multitrait[tiab] AND scaling[tiab] AND (analysis[tiab] OR analyses[tiab])) OR “item discriminant”[tiab] OR “interscale correlation*”[tiab] OR error[tiab] OR errors[tiab] OR “individual variability”[tiab] OR “interval variability”[tiab] OR “rate variability”[tiab] OR (variability[tiab] AND (analysis[tiab] OR values[tiab])) OR (uncertainty[tiab] AND (measurement[tiab] OR measuring[tiab])) OR “standard error of measurement”[tiab] OR sensitiv*[tiab] OR responsive*[tiab] OR (limit[tiab] AND detection[tiab]) OR “minimal detectable concentration”[tiab] OR interpretab*[tiab] OR ((minimal[tiab] OR minimally[tiab] OR clinical[tiab] OR clinically[tiab]) AND (important[tiab] OR significant[tiab] OR detectable[tiab]) AND (change[tiab] OR difference[tiab])) OR (small*[tiab] AND (real[tiab] OR detectable[tiab]) AND (change[tiab] OR difference[tiab])) OR “meaningful change”[tiab] OR “ceiling effect”[tiab] OR “floor effect”[tiab] OR “Item response model”[tiab] OR IRT[tiab] OR Rasch[tiab] OR “Differential item functioning”[tiab] OR DIF[tiab] OR “computer adaptive testing”[tiab] OR “item bank”[tiab] OR “cross-cultural equivalence”[tiab])  NOT  (“addresses”[Publication Type] OR “biography”[Publication Type] OR “case reports”[Publication Type] OR “comment”[Publication Type] OR “directory”[Publication Type] OR “editorial”[Publication Type] OR “festschrift”[Publication Type] OR “interview”[Publication Type] OR “lectures”[Publication Type] OR “legal cases”[Publication Type] OR “legislation”[Publication Type] OR “letter”[Publication Type] OR “news”[Publication Type] OR “newspaper article”[Publication Type] OR “patient education handout”[Publication Type] OR “popular works”[Publication Type] OR “congresses”[Publication Type] OR “consensus development conference”[Publication Type] OR “consensus development conference, nih”[Publication Type] OR “practice guideline”[Publication Type]) NOT (“animals”[MeSH Terms] NOT “humans”[MeSH Terms])  Limits: Full text, human studies, English language |

**S1- Search Strategy**

| **Measurement Property** | **Definition** | **Assessment Criteria** |
| --- | --- | --- |
| Content validity | The degree to which the content of a PROM is an adequate reflection of the construct to be measured | Items of the PROM are relevant, comprehensive, and comprehensible. PROM development study, the quality and results of additional content validity studies on the PROMs (if available), and a subjective rating of the content of the PROMs by the reviewers is taken into account |
| Structural validity | The degree to which the scores of a PROM are an adequate  reflection of the dimensionality of the construct to be measured | Confirmatory Factor Analysis (CFA): Comparative fit index (CFI) or Tucker‐Lewis index (TLI) or comparable measure >0.95 or root mean square error of approximation (RMSEA) <0.06 |
| Internal consistency reliability | The degree of the interrelatedness among the items | At least low evidence for sufficient structural validity AND Cronbach's alpha(s) ≥ 0.70 for each unidimensional scale or subscale |
| Cross-cultural validity/measurement invariance | The degree to which the performance of the items on a  translated or culturally adapted PROM are an adequate reflection  of the performance of the items of the original version of the PROM | No important differences found between group factors (such as age, gender, language) in multiple group factor analysis |
| Test-retest reliability | The extent to which scores for patients who have not changed  are the same for repeated measurement over time | Intraclass correlation coefficient (ICC) ≥0.70 |
| Measurement error | The systematic and random error of an individual patient’s  score that is not attributed to true changes in the construct to be measured | Smallest detectable change (SDC) or limits of agreement (LoA) <minimal important change (MIC) |
| Construct validity | The degree to which the scores of a PROM are consistent with  hypotheses (for instance with regard to internal relationships,  relationships to scores of other instruments, or differences  between relevant groups) | The result is in accordance with the hypothesis |
| Responsiveness | The ability of a PROM to detect change over time in the construct to be measured | The result is in accordance with the hypothesis |
| Interpretability | The degree to which one can assign qualitative meaning ‐ that is, clinical or commonly understood connotations – to a PROM’s  quantitative scores or change in scores | COSMIN criteria for interpretability for not available; assessed by investigating whether a guide to meaningful interpretation of scores was available and graded as ‘available’/‘not available’ |

**S2. Definition and criteria for each measurement property**

Measurement properties and criteria as defined by COSMIN manual for systematic reviews of PROMs [14]

| **PROM**  **(Ref)** | **Structural Validity** | | **Internal Consistency Reliability** | | **Test-Retest Reliability** | | **Construct Validity** | | | **Responsiveness** | | | **Interpretability** | | |
| --- | --- | --- | --- | --- | --- | --- | --- | --- | --- | --- | --- | --- | --- | --- | --- |
|  | **N** | **Extracted results of measurement property** | **N** | **Extracted results of measurement property** | **N** | **Extracted results of measurement property** | **N** | **Extracted results of measurement property** | **N** | | **Extracted results of measurement property** | **N** | | **Extracted results of measurement property** |  |
| QLQ-CIPN15  (Smith et al 2018) | 104 | CFA not confirmed for 2 previously described structures (upper/lower and sensory/motor) | 104 | Cronbach's α for sum score was 0.91, item-item correlations were 0.46-0.81 | 105 | Completed PROM 1-2 hours apart. Correlations r=0.86; CI=0.80-0.90 | 103 | Correlations between TNSc to CIPN15 sum score r =0.57. TNSc subjective and motor item scores also correlated with CIPN15 (r=0.57, r=0.72).  Significant differences in CIPN15 between patients (mean=14.27, SD=17.33) and controls (mean=0, SD=0) | 104 | | ES for CIPN15 small-medium and clinically significant (d=0.52, CI=0.25-0.79) |  | |  |  |
| QLQ-CIPN20  (Cavaletti et al 2013) |  |  |  |  | 264 | Completed PROM 2-3 weeks apart. Good correlations for sensory (r=0.836) motor (r=0.844) and autonomic (r=0.726) subscales |  |  |  | |  |  | |  |  |
| QLQ-CIPN20  (Postma et al 2005) |  |  | 44 | Cronbach's α for sensory, motor and autonomic subscale was 0.82, 0.73 and 0.76. |  |  |  |  |  | |  |  | |  |  |
| QLQ-CIPN20  (Yeo et al 2019) |  |  |  |  |  |  |  |  |  | |  | 343 | | Using distribution-based estimations, MCID for sensory subscale is 2.5-5.9, motor is 2.6-5.0 |  |
| QLQ-CIPN20  (Kieffer et al 2017) | 1254 | CFA on hypothesized models (sensory/ motor/ autonomic, upper/lower extremities) did not fit samples |  |  |  |  | 500-754 | Significant differences between QLQ-CIPN20 and NCI motor and sensory grades (P<0.01).  Significant difference in EORTC-CIPN20 scores in patients vs. controls (P<0.001) |  | |  |  | |  |  |
| QLQ-CIPN20  (Lavoie Smith et al 2013) | 316 | CFA shows poor model fit for sensory/motor/autonomic structure | 321-365 | Cronbach's α for sensory, motor and autonomic scales were 0.88, 0.88 and 0.78. Item to score correlations ranged 0.44-0.63 |  |  | 249 | Correlations between subscales to the CTCAE sensory scale were low (-0.2, 0.2, 0.3). Pain items measure had low-mod correlations to BPI-SF pain questions (r=0.30-0.57, P<0.001). CIPN16 did not correlate with CTCAE (r=0.16-0.21, P<0.05)  Significant difference between patients and controls (P<0.0001) | 173 | | After 12 weeks of chemotherapy treatment, ES on sensory and motor scale were 0.82 and 0.48 |  | |  |  |
| QLQ-CIPN20- Korean Version  (Kim et al 2014) | 249 | CFA found significant model fit for sensory, motor and autonomic substructures | 249 | Cronbach's α for the scale, as well as sensory, motor and autonomic subscales were 0.88, 0.89, 0.88 and 0.73 |  |  | 249 | Significant differences between ECOG PS groups in all sensory, motor and autonomic subscales (all P<0.001). Groups with poor physical activity had higher CIPN scores |  | |  |  | |  |  |
| QLQ-CIPN20-Arabic version  (Abu Sharour et al 2019) | 100 | EFA suggested sensory, motor and autonomic subscales had loadings of 0.68-0.91, 0.68-0.91 and 0.69-0.81 | 100 | Cronbach’s α for sensory, motor and autonomic subscales and total measure were 0.84, 0.79, 0.81 and 0.83 |  |  | 100 | Arabic EORTC-CIPN20 was significantly correlated to Arabic FACT/GOG-Ntx (r=-0.88) and QLQ-C30 (r=-0.65), both P<0.01 |  | |  |  | |  |  |
| QLQ-CIPN20 Electronic and paper/pencil versions  (Knoerl et al 2017) |  |  | 23 | Cronbach's α for motor and sensory subscales for electronic version were 0.76 and 0.75, autonomic items were not correlated with one another. Paper/pencil α were 0.79 and 0.75, with no correlation for autonomic items |  |  | 23 | The sensory and motor subscales of the paper/pencil and electronic versions were significantly correlated to both PRO-CTCAE items |  | |  |  | |  |  |
| QLQ-CIPN16/20  (Smith et al 2019) | 946 | CFA of 3-factor structure showed poor model fit | 1155 | Cronbach's α for the sensory, motor and autonomic (male/female) subscales for CIPN20 were 0.87, 0.83 and 0.62/0.39. CIPN16 two-item factor scale of 0.90 and 0.85 |  |  |  |  |  | |  |  | |  |  |
| QLQ-CIPN20 RASCH model-based testing  (Smith et al 2019) | 1008 | Cluster analysis findings suggested lack of clear factor structure based on sensory, motor and autonomic subscales |  |  |  |  | 1008 | Significant differences between responses of males and females (P<0.0001) and when comparing patients in different chemo groups |  | |  |  | |  |  |
| QLQ-CIPN20 Chinese Version  (Cheng et al 2019) | 212 | CFA found poor model fit for 3-factor structure of CIPN20 | 212 | Cronbach's α of 0.82-0.90 at all assessment timepoints |  |  | 212 | CIPN20 and correlated to WHO-CIPN from cycle 5 to 9months F/U (r=0.40-0.44).  CIPN20 and NCI sensory not significantly correlated at baseline and cycle 1, became significant thereafter.  Significant differences in CIPN20 scores between younger and older (±60 years) at most timepoints except cycle1-3 | 212 | | CIPN20 showed small-mod ES in detecting change throughout treatment progression (r=0.09-0.46) |  | |  |  |
| FACT/GOG-Ntx11  (Huang et al 2007) |  |  | 70-116 | Cronbach's α were 0.8-0.85 for total scale.  Sensory subscale was 0.73-0.91 |  |  | 70-116 | Patients on neurotoxic treatment reported higher scores. | 70-116 | | Fitted linear mixed model estimate indicated that scores increased significantly (P<0.001) from 3.67 at baseline to 8.13 pre cycle 7 |  | |  |  |
| FACT/GOG-Ntx12/13  (Kopec et al 2006) | 304 | EFA demonstrated scale is unidimensional | 304 | Cronbach's α was 0.85 for total scale |  |  | 304 | Correlation with NCI-Sanofi was 0.53 | 304 | | Mean scores increased during chemotherapy from baseline to cycle 2, and at 6 months |  | |  |  |
| FACT/GOG-Ntx11  (Calhoun et al 2003) |  |  | 14-56 | Cronbach's α exceeded 0.70 in 11 of 12 assessments |  |  | 14-56 | Significant difference in PROM score between patients and controls at baseline, 3 and 6 months F/U | 14-56 | | No significant difference in FACT/GOG-Ntx score between patients with improved, stable or declined quality of life |  | |  |  |
| FACT/GOG-Ntx11  (Cella et al 2003) |  |  | 171-196 | Cronbach's α ranged 0.82-0.86 |  |  |  |  | 143 | | Compared to baseline, ES at 6 weeks was 0.4 and 12 weeks was 1.01 |  | |  |  |
| FACT/GOG-Ntx11 Chinese version (Cheng et al 2020) | 118-343 | CFA indicated that fit indices did not meet standard of fit criteria at each timepoint | 118-343 | Cronbach's α ranged 0.82-0.89 from baseline to 12months F/U.  Cronbach's α were stably adequate for three of the four domains (0.64-0.90) |  |  | 118-343 | FACT/GOG showed mod-high associations with EORTC-CIPN20 at all timepoints (r=0.79-0.93), low-mod correlations to NCI sensory (r=0.23-0.45 and NCI motor (r=0.15-0.5) | 118-343 | | Estimated marginal means of scores significantly decreased over time (Wald chi-square- 113.6, P<0.001) | 118-343 | | Distribution-based method yielded MCID values of 1.38-2.21 using 0.3SD and 2.30-3.68 using 0.5SD |  |
| GOG-NtxX11  (Almadrones et al 2004) | 67-88 | EFA anticipated two-factor structure (upper/lower). All items loaded clearly on one factor  or the other | 67-88 | Cronbach's α ranged 0.89-0.91 |  |  |  |  | 67-88 | | Significant changes existed in between pre-treatment and end of treatment |  | |  |  |
| FACT/GOG-Ntx Chinese Version  (Cheng et al 2019) | 212 | Three factor structure of FACT/GOG-Ntx not well supported | 212 | Cronbach's α of 0.82-0.91 at all assessment timepoints |  |  | 212 | FACT/GOG moderately correlated to WHO-CIPN from cycle 5 to 9months F/U (r=-0.42 to -0.46).  FACT/GOG moderately associated with NCI sensory from cycle 3 to 12months F/U (r=0.44-0.55).  Age differences were identified at 3 timepoints | 212 | | Small-moderate ES in detecting change throughout treatment progression (r= 0.11-0.35) |  | |  |  |
| FACT/GOG-Ntx  (Alberti et al 2021) |  |  |  |  |  |  |  |  | 214 | | Significant score changes from baseline to end of treatment (ES= 2.47, P<0.001) |  | |  |  |
| CIPNAT  (Tofthagen et al 2011) |  |  | 126 | Cronbach’s α for total CIPNAT was 0.95, symptom experience items 0.93 and interference items 0.91 | 30 | Test-retest scores were all highly correlated for entire measure (r=0.93, P<0.001), symptom experience (r=0.89, P<0.001) and interference items (r=0.93, P<0.001) | 167 | CIPNAT and FACT/GOG were highly correlated (r=0.83, P<0.001).  Mean scores between patients and controls were significantly different overall, and for 2 item sets of CIPNAT |  | |  |  | |  |  |
| CIPNAT-Turkish Version  (Simsek et al 2018) | 282 | PCA conducted, and structures (disturbance, emotional, frequency, disturbance in daily life) yielded factor powers of 0.553-0.937 | 282 | Cronbach's α was 0.87 for whole scale, structures ranged 0.87-0.97 | 122 | Re-test 2 weeks later showed strong correlation between tests (ranged 0.9-0.93 for each subscale) |  |  |  | |  |  | |  |  |
| CIPNAT- Turkish Version  (Kutluturkan et al 2017) | 327 | CFA was conducted and verified structure of PROM | 327 | Cronbach's α for entire scale is 0.971. Coefficients for sensory, motor, fine motor activities and general activities subscales ranged 0.81-0.97 | 40 | Re-test at 2 weeks had correlation r=0.89, P<0.001 | 327 | CIPNAT subscales were all significantly correlated to CIPN20 subscales (sensory, motor, autonomic) (r=0.25-0.85, P<0.001) |  | |  |  | |  |  |
| TNASv1, v2  (Mendoza et al 2015) |  |  | 409 | Cronbach's α was 0.86-0.87 for whole scale, 0.80-0.85 for sensory and motor subscales |  |  |  |  | 164 | | Comparing pre and post treatment, in colorectal cancer cohort, 10/11 TNAS items significantly worsened. Multiple myeloma had 4/11 items worsened.  ES for each item ranged 0.04-2.12 |  | |  |  |
| TNASv3  (Mendoza et al 2020) |  |  | 60 | Cronbach's α was 0.88 and 0.9 for first and second administration | 60 | ICC=0.97  First test was completed onsite, second at home |  |  |  | |  |  | |  |  |
| CAS-CIPN  (Kanda et al 2019) | 327 | EFA using principal factor method, sampling validity was 0.821. 4 items were removed for high factor loading. Convergence was reached with 4 factors comprising 15 items | 327 | Cronbach's α was 0.826 for entire scale. Subscales ranged 0.757-0.860 |  |  | 87 | Participants divided between high and low scoring groups on total FACT/GOG scores. There was a significant difference of 22.6 points on CAS-CIPN (P<0.001) |  | |  |  | |  |  |
| ICPNQ  (Beijers et al 2016) |  |  | 156 | Cronbach’s α for sensory and motor subscales were 0.84 and 0.74 and α of autonomic scale was moderate (0.61). | 117 | Good test- retest reliability after 1 month ICC=0.83 (CI 0.76–0.89), 0.83 (CI 0.75–0.89), and 0.77 (CI 0.67–0.84) for sensory, motor, and autonomic scales. Test-retest for the defined ICPNQ grades were 0.67 (CI 0.53–0.77) | 156 | All subscales correlated with CIPN20 (r=0.40- 0.72). ICPNQ able to distinguish patients who did/did not receive chemotherapy (P=0.006) and who had a lower physical performance status (P=0.001) |  | |  |  | |  |  |
| K-NTX-4  (Lee et al 2019) |  |  | 237 | Cronbach's α was 0.89 | 190 | Re-test at 7-21 days later ICC=0.84 | 237 | Weak correlation between K-NTX-4 and Korean National Comprehensive Cancer Network (NCCN)–  Functional Assessment of Cancer Therapy (FACT) Ovarian  Symptom Index-18 (K-NFOSI-18; r=0.348). Weak-mod correlation to Korean version of the Euro-  QoL-5 Dimension (K-EQ5D), r=0.2-0.5 |  | |  |  | |  |  |
| CIPN R-ODS  (Binda et al 2013) |  |  | 281 | Pearson separation index (PSI) value was high (0.92) | 281 | Re-test 2-3 weeks later, acceptable test-retest reliability- items' hierarchy and patients' ability location were mainly within 95% CI |  |  |  | |  |  | |  |  |
| OANQ/CINQ  (Gustafsson et al 2016) |  |  | 23 | Cronbach α for total as well as three domains (upper/lower/oral) were 0.84-0.94 | 24 | Re-test 1 hour later, no significant differences for each of 3 domains.  ICC showed 69% of symptoms had excellent reproducibility, 24% was fair-good and 7% were poor |  |  |  | |  |  | |  |  |
| PRO-CTCAE  (Dueck et al 2015) |  |  |  |  | 80 | Re-test 1 day later. ICC for severity is 0.8, interference is 0.55 | 846 | Weak-moderate correlations with physical functioning, role functioning, emotional functioning, social functioning, global health Status/QoL (r=0.11-0.32) |  | |  |  | |  |  |
| PRO-CTCAE  (McCrary et al 2019) |  |  |  |  |  |  | 644 | PRO-CTCAE moderate-highly correlated to TNSr (r=0.56) and FACT-GOG (r=0.75).  TNSr and FACT/GOG significantly different between grades on PRO-CTCAE (P>0.05).  PRO-CTCAE distinguished between mild/no (G0-1) PN to clinically significant PN (G2-4) |  | |  |  | |  |  |
| PRO-CTCAE  (Knoerl et al 2021) |  |  |  |  | 123 | Re-test after 131 mins. ICC for severity=0.79, interference=0.73 | 142 | PRO-CTCAE highly correlated (Spearman’s ρ=0.72) with QLQ-CIPN20 sensory subscale, and moderately correlated with motor subscale (ρ=0.50). Low correlations between TNSc and PRO-CTCAE (ρ=0.30-0.48) |  | |  |  | |  |  |
| PNQ  (Shimozuma et al 2009) |  |  |  |  |  |  | 300 | Weighted kappa (when compared to NCI) sensory =0.16; motor disturbance =0.02 . PNQ sensory and motor subscales correlated with FACT/GOG (r=0.66 and r=0.51) | 300 | | PNQ sensory and motor scores significantly increased as treatment cycles increased (P<0.0001) |  | |  |  |
| 10-Point VAS  (Takemoto et al 2011) |  |  |  |  |  |  |  |  | 59 | | VAS score for numbness significantly increased from 1-2 and 3-4 cycles |  | |  |  |
| CIPN Self check sheet  (Miyoshi et al 2014) |  |  |  |  |  |  | 77 | Compared to self-check sheet, free-style interview not able to capture grade 3 symptoms. Self-check sheet able to capture all grade 3 cases, had k coefficient of 0.988 p<0.01 |  | |  |  | |  |  |

**S3- Measurement property results extraction**

*Abbreviations: CFA confirmatory factor analysis, CI confidence interval, EFA exploratory factor analysis, ES effect size, F/U Follow-up, ICC intraclass correlation coefficient, MCID minimal clinically important difference, NCI National Cancer Institute Common Terminology for Adverse Events Neuropathy Sensory subscale, PCA principle component analysis, PN peripheral neuropathy, PROM patient reported outcome measure, SD standard deviation, VAS visual analogue scale*
